# Effect of Natural Nanostructured Rods and Platelets on Mechanical and Water Resistance Properties of Alginate-Based Nanocomposites

**DOI:** 10.3389/fchem.2018.00635

**Published:** 2018-12-19

**Authors:** Dajian Huang, Zhuo Zhang, Zonghong Ma, Qiling Quan

**Affiliations:** School of Materials Science and Engineering, Lanzhou Jiaotong University, Lanzhou, China

**Keywords:** palygorskite, montmorillonite, bionanocomposite films, sodium alginate, mechanical strength, water resistance

## Abstract

A series of biopolymer-based nanocomposite films were prepared by incorporating natural one-dimensional (1D) palygorskite (PAL) nanorods, and two-dimensional (2D) montmorillonite (MMT) nanoplatelets into sodium alginate (SA) film by a simple solution casting method. The effect of different dimensions of nanoclays on the mechanical, water resistance, and light transmission properties of the SA/PAL or MMT nanocomposite films were studied. The field-emission scanning electron microscopy (FE-SEM) result showed that PAL can disperse better than MMT in the SA matrix in the case of the same addition amount. The incorporation of both PAL and MMT into the SA film can enhance the tensile strength (TS) and water resistance capability of the film. At a high content of nanoclays, the SA/PAL nanocomposite film shows relatively higher TS, and better water resistance than the SA/MMT nanocomposite film. The SA/MMT nanocomposite films have better light transmission than SA/PAL nanocomposite film at the same loading amount of nanoclays. These results demonstrated that 1D PAL nanorods are more suitable candidate of inorganic filler to improve the mechanical and water resistance properties of biopolymers/nanoclays nanocomposites.

## Introduction

Over the past decades, the commodity plastics (i.e., polyethylene, polypropylene, and polyethylene terephthalate) as commonly used food packaging materials have plaid very important roles in human daily production. However, these plastic packaging materials are totally non-biodegradable, so their widespread use caused serious environmental pollution problems (Souza et al., 2017; Costa et al., 2018; Salama et al., 2018). Therefore, the development of biodegradable films using natural, non-toxic, and environment benign polymers such as polysaccharides, proteins, and lipids has drawn much more attention in both of academic and industrial areas (Mushi and Berglund, 2014; Wang and Jing, 2017; Youssef and El-Sayed, 2018). Among numerous biodegradable natural polymers, sodium alginate (SA) was especially concerned owing to its excellent biocompatibility, film-forming ability, and active functional groups (Shankar et al., 2016; Fabra et al., 2018). SA is an anionic natural biomacromolecule, which is composed of poly-b-1, 4-D-mannuronic acid (M units), and a 1, 4-L-glucuronic acid (G units) in different proportions by 1–4 linkages. It is extracted from marine algae or produced by bacteria, and so it has the advantages including abundance, renewability, non-toxicity, water-solubility, biodegradability, and biocompatibility (Wang and Wang, 2010). However, the inherent hydrophilicity and brittleness of neat SA films limited their applications in film materials (Rhim, 2004; Zhang et al., 2017).

In order to overcome the drawbacks of neat SA films, a variety of nanoscale particles such as montmorillonite (MMT) (Tunç and Duman, 2010; Zlopasa et al., 2015), graphene oxide (Liu et al., 2017), and cellulose nanocrystals (Sirvio et al., 2014) have been incorporated into the SA matrix to fabricate a nanocomposite. Abdollahi et al. (2013) developed an alginate/MMT nanocomposite by a solvent casting method, and found that the mechanical properties of the alginate/MMT composites were enhanced significantly after the addition of MMT. However, MMT forms an agglomeration in the polymer matrix when its addition amount exceeds a certain value, which leads to the decrease of the mechanical properties of the film. It has been shown that MMT is a 2:1-type layered clay mineral with a sandwiched structure composed of two 2D platelets and interlayer cations (i.e., Na^+^, Ca^2+^, Mg^2+^). The strong hydrogen-binding and electrostatic interaction, and van der Waals forces between two platelets make MMT difficult to be exfoliated and tend to be present in a form of agglomeration (Zhang et al., 2014; Block et al., 2015; Liu et al., 2016). In comparison, natural 1D rod-like nanoclays are easy to be dispersed as nanoscale size, and showed great potential to be used to develop polymer/nanoclays composites (Nikolic et al., 2017; Ajmal et al., 2018; Shankar et al., 2018; Zhang P. et al., 2018). It has been demonstrated that the dispersion of nanoclays in polymer matrix, and the comprehensive performance of the resultant polymer composites exhibited interesting dependence on the shape of fillers. Usually, rod-like nanoclays have a relatively smaller contact surface and weaker interaction amount rods, so that they could probably be dispersed in the polymer matrix well with less aggregation (Bilotti et al., 2009). Palygorskite (PAL) is a naturally available 1D nanorod-like silicate clay mineral (Deng et al., 2012; Zhang et al., 2018b). It consists of two double chains of the pyroxene-type (SiO_3_)^2−^ like amphibole (Si_4_O_11_)^6−^ running parallel to the fiber axis (Gard and Follett, 1968; Zhu et al., 2016; Zhang et al., 2018a). PAL is a potential filler to fabricate polymer composite due to its unique advantages, such as high aspect ratio, large specific surface area, good thermal stability, and high modulus (Huang et al., 2012; Ruiz-Hitzky et al., 2013; Ding et al., 2019). It has been confirmed that the incorporation of silylated PAL into the polyurethane matrix improved significantly the thermal stability and mechanical properties of polyurethane (Peng et al., 2011). In addition, 1D fibrous nanoclay has relatively higher density of silanol groups on its surface than 2D layered silicates, making it able to form more hydrogen bonds with hydrophilic biopolymers (Alcantara et al., 2014). So far, the studies on the comparison of 1D and 2D nanoclays in fabricating SA/nanoclays nanocomposites still received less attention.

In this paper, we have prepared a series of SA/nanoclays nanocomposite film using 1D PAL and 2D MMT as the inorganic ingredients, and studied the effect of different dimensions of nanoclays on the structure, organic/inorganic interface interaction and the mechanical, water resistance, light transmission properties of the films. The potential of 1D PAL nanorods and 2D MMT for fabricating SA-based nanocomposite film was also assessed by a systematic comparative study.

## Experiment Section

### Materials

SA [characteristic viscosity of 1% aqueous solution at 20 °C ≥0.02 Pa●s, mannuronic (M) acid/guluronic (G) acid ratio = 65/35, molecular weight ≈120,000 g/mol] was purchased from Sinopharm Chemical Reagent Co., Ltd. (Shanghai, China). Glycerol was obtained from Guangdong Guanghua Chemical Factory Co., Ltd. (Guangdong, China). MMT was purchased from Zhejiang Fenghong New Material Co., Ltd. (Zhengjiang, China). PAL was purchased from Changzhou Dingbang Clay Co., Ltd (Jiangsu, China).

### Preparation of SA/PAL and SA/MMT Nanocomposite Films

The SA, SA/PAL, and SA/MMT films were prepared by a solution casting method. The aqueous solution containing 2 wt.% of SA were prepared by dissolving SA powder into distilled water under mechanical stirring for 24 h. Then, glycerol (40 w/w% of the mass of SA) as a plasticizer was added into the SA solution, and the mixture was stirred continuously at 600 rpm to obtain a homogeneous solution. PAL or MMT dispersions (2 wt.%) were prepared by dispersing given amounts of clays in distilled water under mechanical agitation at 600 rpm for 1 h at room temperature. Afterward, the obtained mixture was dispersed with the aid of ultrasound equipment operating at 40 kHz for 10 min. The obtained dispersion of PAL or MMT was added to the SA solutions (the amount of clays is 2, 4, 6, 8, and 10 wt.% of the mass of SA) to form a precursor solution for film-forming. Then, the resultant mixture solutions were poured into the PS dishes (9 cm diameter) and evaporated at atmosphere for 72 h. After drying, the films were peeled off from the plate surface and then kept in a conditioning cabinet with the relative humidity (RH) of 53% for further treatments. The neat SA film was prepared according to a similar procedure without the addition of PAL or MMT.

### Test of Mechanical Properties

The tensile performance of the film with length of 80 mm and width of 10 mm was studied using an AG-IS material testing machine (Shimadzu Co., Ltd., Japan). A 200 N load cell was used, and the strain rate was 10 mm/min. Samples were studied in each test and all tests occurred at room temperature. The averages of tensile strength and elongation at break of five specimens of each film were taken and presented.

### Test of Moisture Uptake

The moisture uptake (MU) was measured by cutting the film samples into small pieces (2 × 2 cm). The samples were dried at 70°C for 24 h. After the samples were weighed (W_0_), they were conditioned for 72 h at 76% RH (saturated NaCl solution) to ensure equilibrium of the moisture before reweighing (W_1_).

MU of the samples was calculated as follows:

(1) $$\begin{array}{r} \text{MU}=\left[{\text{W}}_{1}-{\text{W}}_{0}\right]/{\text{W}}_{0}\times 100\% \end{array}$$

An average value of five replicates for each sample was taken.

### Characterizations

The crystalline structure of samples was characterized with an X-ray diffractometer (Philips, X'pert PRO, The Netherland), with the Cu Ka radiation at 40 kV and 30 mA. The diffraction patterns were collected in reflection mode by scanning the 2θ range from 5° to 30°, with a scan speed of 6°/min. A FTIR Spectrophotometer (Thermo Nicolet, NEXUS 670, USA) was employed to record the Infrared spectra of films with the attenuated total reflectance (ATR) model in the range of 4,000–500 cm^−1^. The surface morphology of samples was observed with a JEOL JSM-6701F microscope after the samples were coated with gold. The films for test were immersed in liquid nitrogen and cryo-fractured manually. TEM images of samples were taken with a TECNAIG2-F30 Transmission electron microscope (FEI, USA). Light transmission of films was measured with a UV–vis spectrophotometer [N4, INESA (Group) Co., Ltd, China] at selected wavelengths between 200 and 600 nm.

## Results and Discussion

### XRD Analysis

**Figure 1:**
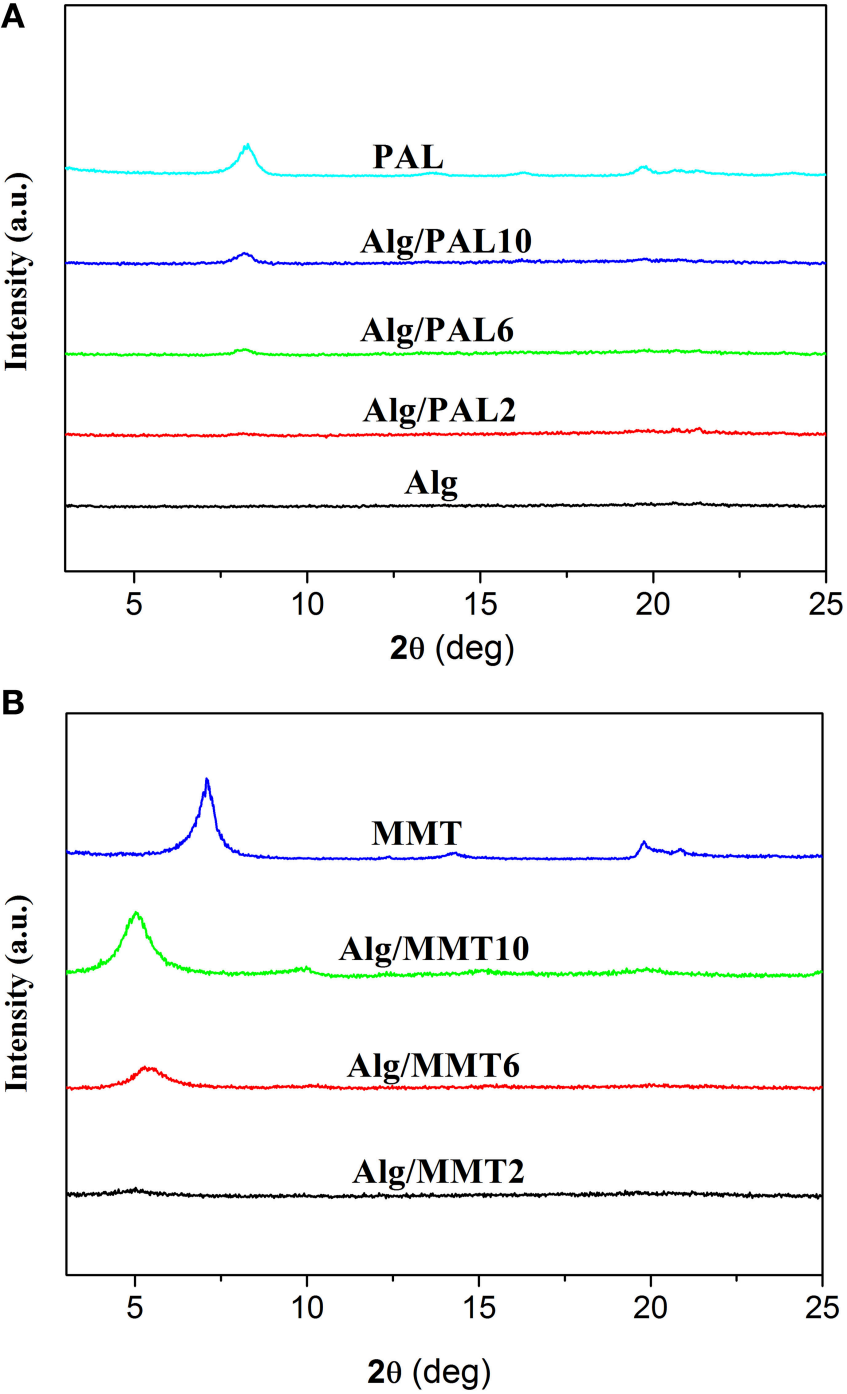
XRD patterns of **(A)** PAL, SA film, and SA/PAL nanocomposite films; and of **(B)** MMT and SA/MMT nanocomposite films.

As shown in Figure 1, no sharp diffraction peaks were observed in the XRD patterns of SA/glycerol films (Figure 1A), indicating that neat SA film shows no crystalline state and the polymer chains are arranged randomly. The neat PAL showed the diffraction peak at 2θ = 8.4°, ascribed to the characteristic reflection of (110) crystallographic plane (Figure 1A) (Xu et al., 2017). The diffraction peak of (110) plane of PAL (2θ = 8.4) was observed in the XRD pattern of SA/PAL nanocomposite films. The position of (110) diffraction peak has not been changed, but the peak intensity increased with increasing the loading amount of PAL in the SA/PAL film. This result indicated that SA polymer chains cannot enter the tunnel to cause the change of the crystal structure of PAL, which is in good agreement with previous reports (Peng et al., 2011; Huang et al., 2012). Different from smectite, PAL has a layer-chain structure in which the structure unit composed of continuous tetrahedrons and discontinuous octahedrons are very stabile and cannot be intercalated or exfoliated, so the crystal structure, and morphology of PAL can remain well after forming nanocomposite.

The XRD patterns of MMT and the SA/MMT nanocomposite films with different loading amount of MMT are presented in Figure 1B. An intense diffraction peak at 2θ = 7.06° (basal spacing is 1.25 nm), ascribed to the characteristic reflection of (001) crystalline plane of MMT, was observed in the XRD pattern of MMT. The characteristic reflection of MMT has not been observed in the XRD pattern of SA/MMT when the content of MMT is 2 wt.%, indicating MMT was exfoliated after its composite with SA polymer chains at a relatively lower MMT loading amounts. When the loading amount of MMT increased to 6 and 10 wt.%, the diffraction peak of MMT shifted from 7.06° to 5.28° and 5.02°, respectively. These results indicated that some SA polymer chains were intercalated into the interlayer space of MMT (Majdzadeh-Ardakani and Nazari, 2010), forming an intercalated-exfoliated nanocomposite structure at relatively higher contents of MMT (Nagarajan et al., 2015).

### FTIR Analysis

**Figure 2:**
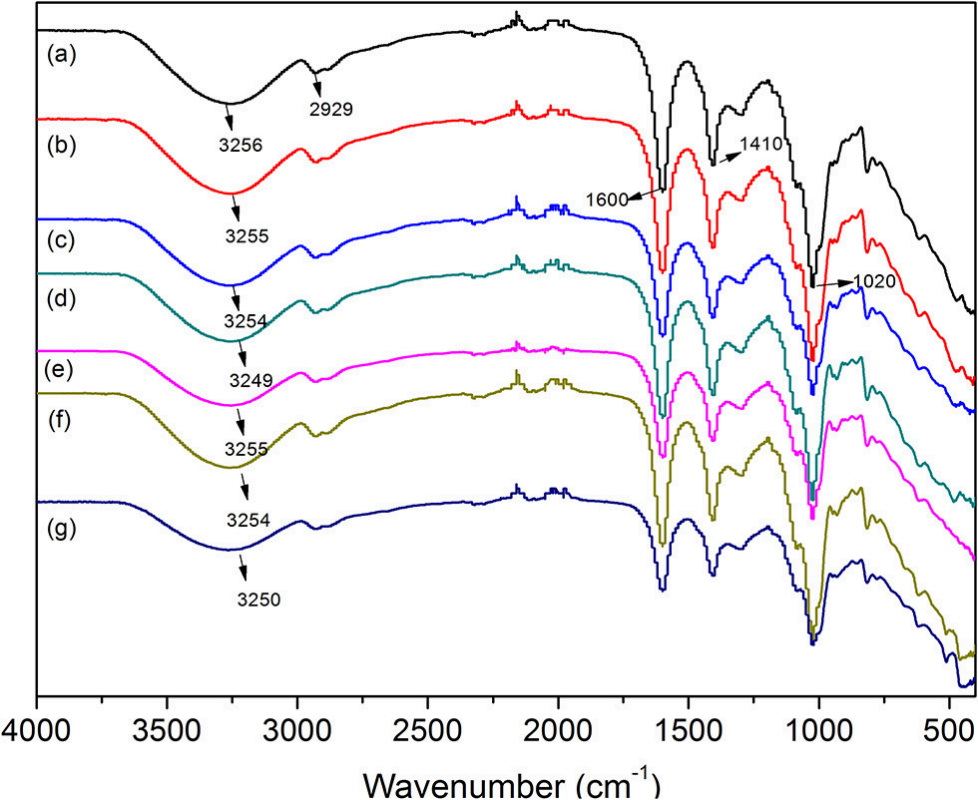
ATR-FTIR spectra of **(A)** neat SA film, **(B)** SA/PAL2, **(C)** SA/PAL6, **(D)** SA/PAL10, **(E)** SA/MMT2, **(F)** SA/MMT6, and **(G)** SA/MMT10.

The incorporation of PAL or MMT into SA matrix was evaluated by ATR-FTIR analysis of films, because the vibrations and shifts of the FTIR absorption bands may reflect the interactions between SA and nanoclays. The ATR-FTIR spectra of the SA and SA/PAL or SA/MMT nanocomposite films are shown in Figure 2. As shown in Figure 2A, the absorption bands of neat SA film at 3,256, 1,600 and 1,410, 1,020 cm^−1^ can be attributed to the O-H stretching vibration, the COO symmetric, and asymmetric stretching vibration of carboxylate groups, and the C-O-C stretching vibration (Tezcan et al., 2012). After introducing PAL or MMT into the SA matrix, the absorption band at 3,256 cm^−1^ shifted to low wavenumber region (Figures 2B–G), which confirmed there are strong interaction between SA and PAL or MMT, and the incorporation of PAL or MMT partially break the the hydrogen bonding between SA and glycerol, and formed new hydrogen bonding between SA chains and PAL or MMT (Huang et al., 2004; Liu M. et al., 2012). A similar tendency has also been observed in the CS/PVA/PAL composite film (Huang et al., 2012).

### Micrographs of PAL, MMT, and Corresponding Composites

**Figure 3:**
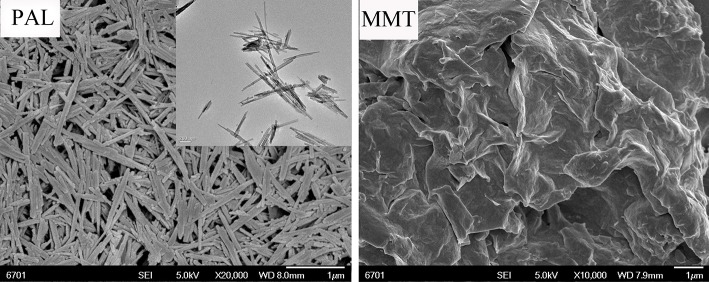
SEM and TEM micrographs of PAL and SEM micrograph of MMT.

Figure 3 showed the SEM and TEM micrographs of PAL and the FESEM image of MMT used in this study. It can be seen from the SEM and TEM images of PAL that the PAL shows good rod-like shape with a diameter of 30–70 nm, and a length of about 0.3–1.5 μm. The rods are slightly aggregated together to form a loose bundle. The MMT exhibits a platelet-like shape, but most of the lamellae of MMT stacked together without presence of single MMT layers.

**Figure 4:**
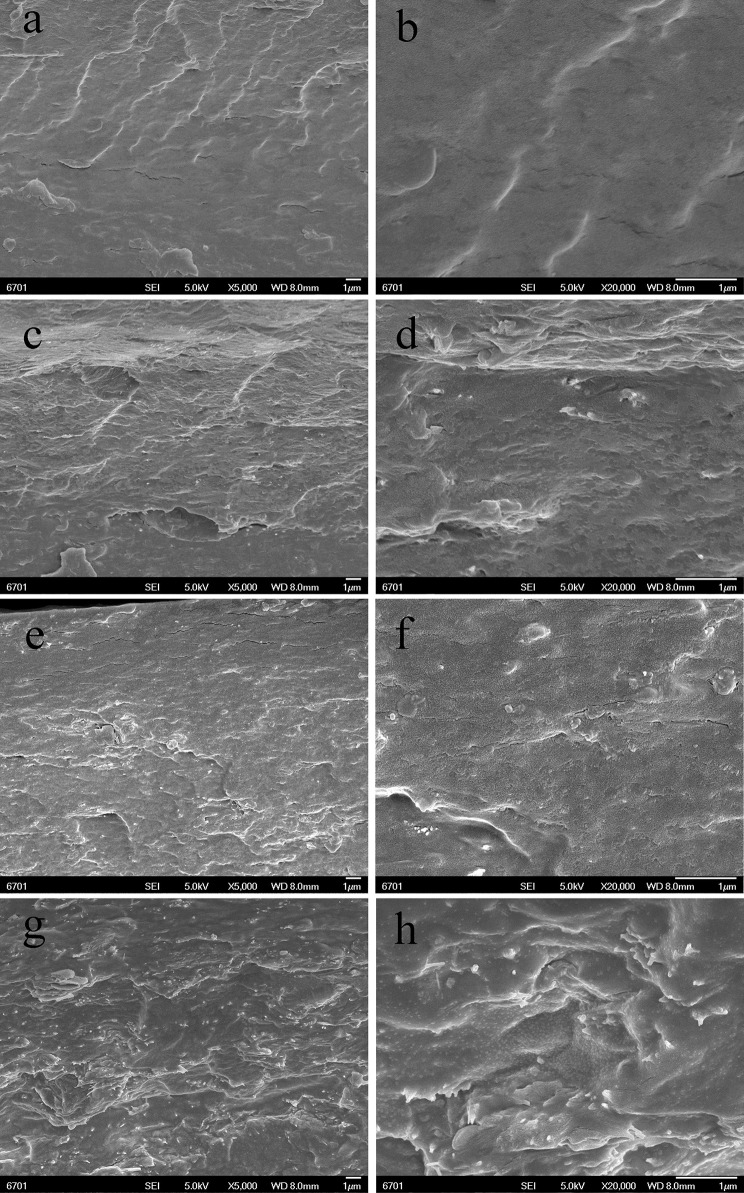
SEM micrographs of the cross-sections of SA/PAL nanocomposite films: neat SA films **(A,B)**, SA/PAL (4 wt.%) **(C,D)**, SA/PAL (6 wt.%) **(E,F)**, and SA/PAL (10 wt.%) **(G,H)**.

The dispersion of the PAL or MMT in the SA matrix was also studied by SEM observation. The SEM images (magnifications × 5,000 and × 20,000) of the cross-section of SA/PAL nanocomposites with the PAL loading of 0, 2, 6, and 10 wt.% are shown in Figure 4, respectively. The fracture surface of neat SA film showed a glossy and ordered morphology, indicating the neat SA film has homogeneous microstructure, and the SA and glycerol are compatible very well. All the SA/PAL nanocomposites showed much rougher fractured surfaces in comparison with SA film. The bright dots observed in the SEM image of the nanocomposites indicated that the PAL rods embedded within the SA matrix very well. The number of the bright dots reflecting a well-dispersion of inorganic components increased with increasing the loading amount of PAL. The PAL has a well-dispersion in the SA matrix without obvious agglomeration, indicating a good compatibility between SA matrix and PAL. The well-dispersion of PAL in the SA matrix can be ascribed to the following reasons. First, the 1D nanorod-like feature of PAL and the relatively weaker interaction among rods allowed them easy to be dispersed in polymer matrix under the action of shear forces. Secondly, PAL can interact with SA via hydrogen bonding interactions, which is also helpful to strip effectively the crystal bundles as individually dispersed nanorods. In addition, the good compatibility between PAL and SA matrix also indicated a strong matrix-filler interfacial adhesion, which facilitates to enhance the mechanical properties of the resulting SA/PAL composite films.

**Figure 5:**
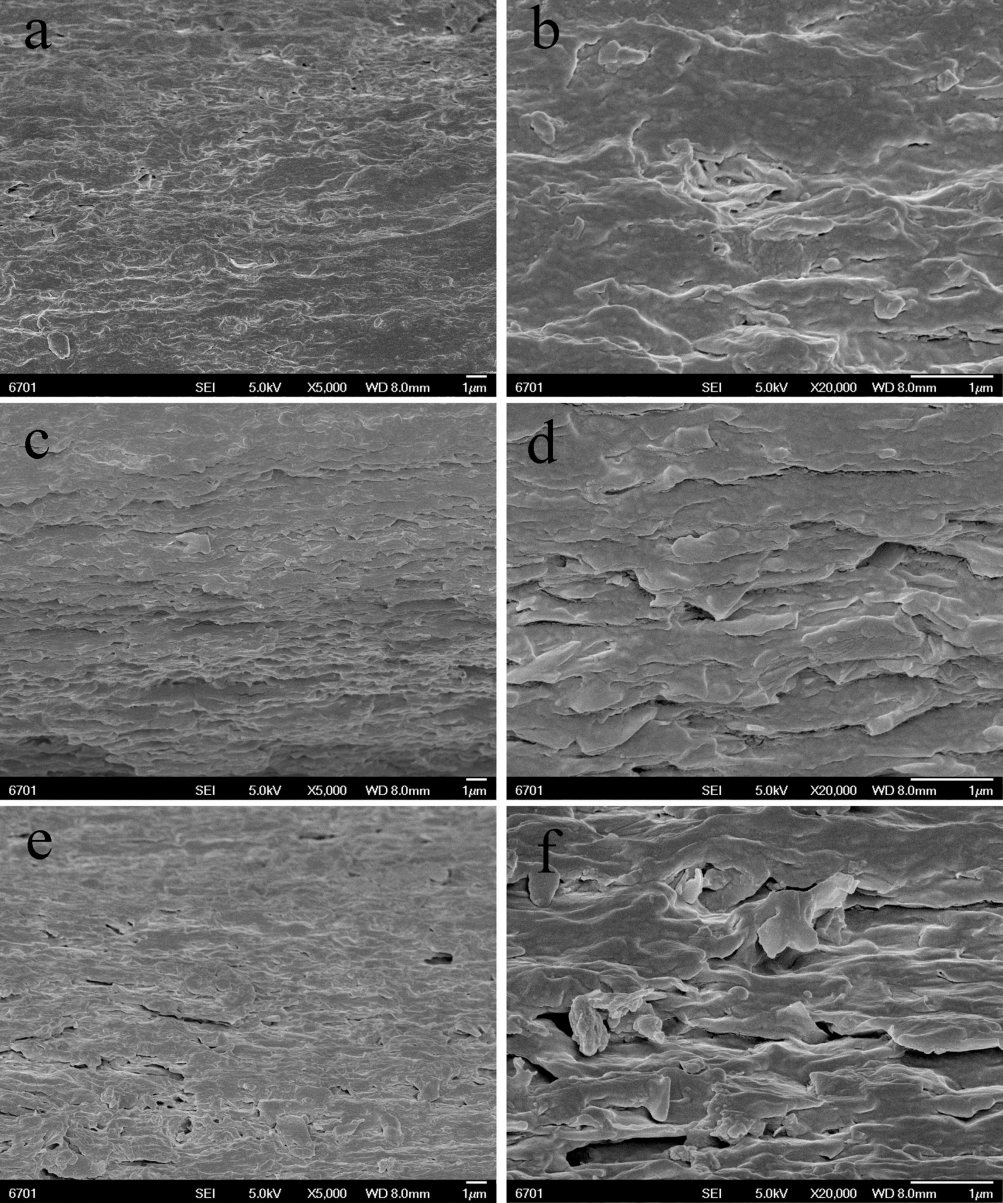
SEM images of the surface of SA/MMT nanocomposite films: SA/MMT (2 wt.%) **(A,B)**, SA/MMT (6 wt.%) **(C,D)**, and SA/MMT (10 wt.%) **(E,F)**.

Figure 5 showed the SEM micrographs of the cross-section of SA/MMT nanocomposite films with the MMT loading amounts of 2, 6, and 10 wt.% at different magnifications (× 5,000 and × 20,000). Compared with the neat SA film, the cross-section of SA/MMT nanocomposite films is more rough and uneven in varying degrees. The roughness degree of cross-section of SA/MMT films increased with increasing the loading amount of MMT, owing to the platelet-shaped structure of MMT. It can be seen that the MMT platelets were directionally stacked and densely packed with SA matrix, forming a laminated structure (Figure 5D). When the loading amount of MMT increased to 10%, the cross-section of SA/MMT nanocomposite film becomes looser owing to the agglomeration of MMT in the SA matrix, which may have a negative impact on the performance of the composites. From Figures 4, 5 it can be seen that PAL has relatively better dispersion than MMT in the SA matrix at high loading amounts (6 and 10 wt.%). This behavior may be because that the interactions among PAL rods are relatively weaker than the interaction between the nanoplatelets of MMT (Lu et al., 2005; Liu M. X. et al., 2012).

### Moisture Uptake (MU)

**Figure 6:**
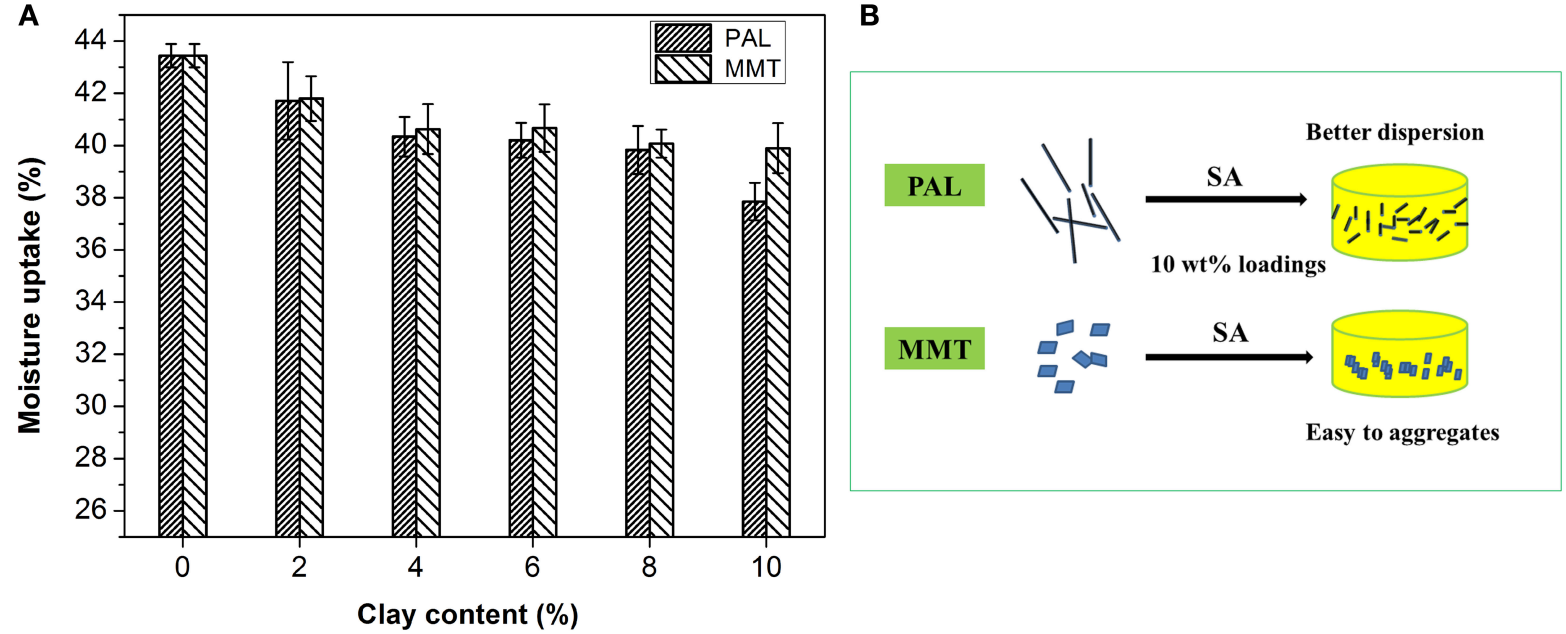
**(A)** MU of the SA/PAL and SA/MMT nanocomposite films with different loadings of nanoclays and **(B)** Schematic illustration of the presence of PAL or MMT in the SA matrix.

MU capability of the SA/PAL and SA/MMT was measured to study the effect of the nanoclays on the water-resistant properties of the SA films. As shown in Figure 6A, The MU of neat SA film was higher than that of SA/PAL and SA/MMT nanocomposites, indicating that the introduction of PAL or MMT can inhibit the penetration of water molecules into the film, and decrease the MU of the nanocomposite films (Almasi et al., 2010). This reduction of MU is because that PAL and MMT are able to form hydrogen bonding networks with SA matrix, increase the surface roughness of film, and block the diffusion pathway of water molecules, which decreased the water sensitivity of the nanocomposites (Wu et al., 2009; Almasi et al., 2010; Bidsorkhi et al., 2014). In addition, the hydrophilicity of PAL or MMT is weaker than that of neat SA matrix, so the incorporation of PAL or MMT is favorable to reduce the MU.

In comparison, the PAL has a relatively better effect than MMT on reducing the MU of SA film. The total MU of SA film reduced about 12.9% after the incorporation of 10 wt.% PAL, which was higher than that of SA/MMT10 (MU only reduced 8.1%). This can be attributed to the fact that PAL has a better dispersion than MMT in the SA matrix at high loadings of nanoclays (see Figure 6B). The good dispersion of PAL nanorod in SA matrix enable it to form more stable and dense hydrogen bond networks with the hydroxyl groups of SA than MMT (little agglomeration in SA matrix) at the same loading amounts. In addition, SA/MMT nanocomposites had a relatively looser surface structure than SA/PAL nanocomposites at the sample loading amount of 10 wt.%, which enable them to absorb easily more water molecules.

### Mechanical Performance of SA/PAL and SA/MMT Nanocomposites

**Figure 7:**
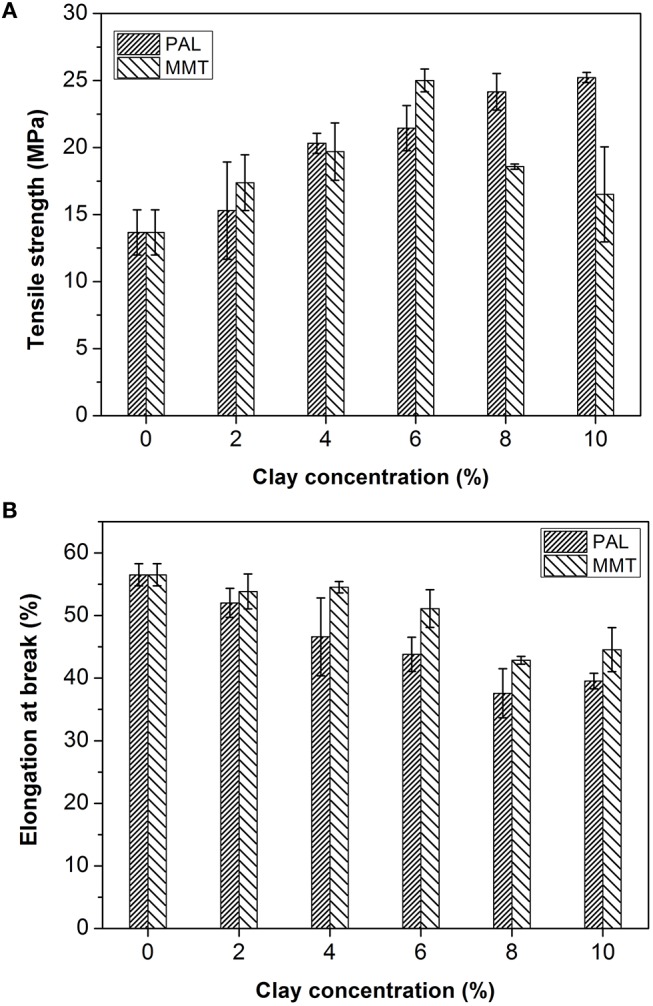
TS **(A)** and EB **(B)** of SA/PAL and SA/MMT nanocomposites with different contents of nanoclays.

The effect of PAL and MMT addition on mechanical properties of SA film was studied by a tensile testing experiment. As shown in Figure 7A, the addition of PAL or MMT has great influence on the tensile strength of nanocomposites. The tensile strength (TS) of SA/PAL nanocomposites increased by 84.56% (from 13.67 to 25.23 MPa) with an increase of PAL loading amount from 0 to 10 wt.%. This obvious improvement of TS of SA/PAL can be ascribed to the homogenous dispersion of PAL in SA matrix and the strong hydrogen bonding interaction between the silanol groups of PAL and the –OH or –COOH groups of SA. In addition, the high aspect ratio of PAL was also favorable to the stress transfer when the film was subject to be stretched. The similar results were observed in other PAL nanorod-reinforced polymers (Chen et al., 2012; Liu et al., 2014).

The TS of SA/MMT increased initially, reached the optimal value at the MMT content of 6 wt.%, and then decreased with the increase of addition amounts of MMT. The TS of nanocomposite films increased by 82.96% in contrast to the SA films. The TS of nanocomposites decreased when the MMT loading amount is larger than 6 wt.%. This tendency is ascribed to the aggregation of MMT in the SA matrix, as confirmed by SEM and XRD analysis results. The similar behavior was also observed by previous research result that the tensile strength of gelatin/MMT films decreased after adding 5% of MMT due to a loss in the quality of MMT dispersion in the gelatin matrix (Flaker et al., 2015).

Figure 7B showed the elongation at breakage (EB) of SA film, SA/PAL, and SA/MMT nanocomposite films. The EB of nanocomposites showed a decreasing trend with the increase of the loading amounts of PAL or MMT. This reduction of EB of SA/PAL and SA/MMT nanocomposite films can be attributed to the fact that the inclusion of rigid clay restricted the motion of SA molecular chains in the matrix. Peng et al. (2011) confirmed that the EB of the waterborne polyurethane/PAL nanocomposite decreased with the increase of PAL loading amount. Slavutsky et al. (2014) also proved the EB of brea gum/MMT composites decreased with increasing the MMT content.

### Light Transmission and Transparency

**Figure 8:**
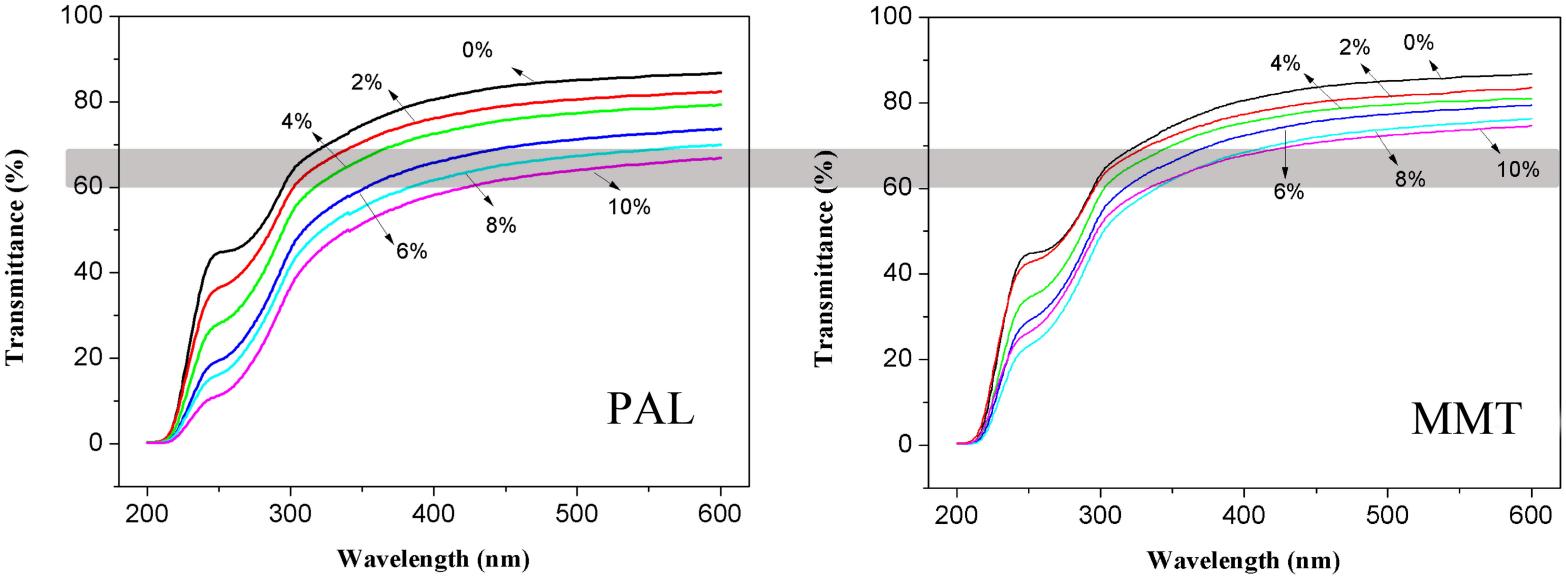
Light transmission properties of SA film, SA/PAL, and SA/MMT nanocomposite films with different contents of nanoclays.

The light transmission of neat SA film, SA/PAL, and SA/MMT nanocomposite films as a function of wavelength is shown in Figure 8. The transmittance of SA film is about 87% at 600 nm (visible region), but it decreased slightly with the increase of PAL or MMT contents. This decrease was possibly related to the presence of PAL or MMT nanoparticles, because light would be absorbed partly by clay nanoparticles, leading to the decrease of energy of transmitted light. This result is similar to the previous findings that the light transmittance of pure polyimide film at 800 nm sharply decreased by incorporating 7 wt.% of PAL (An et al., 2008). Slavutsky et al. (2014) also found that the light transmission of brea gum/MMT composite films decreased after incorporation of MMT.

In addition, the light transmittance of SA/PAL nanocomposites is slightly lower than that of SA/MMT nanocomposites at the same addition amount. The light transmittance of the nanocomposites with addition of 10 wt.% PAL and 10 wt.% MMT at 600 nm was about 67 and 75%, respectively. This result may be due to the diameter of PAL nanorods (50–100 nm) is higher than the thickness of MMT nanoplatelets (about 1 nm) (Ge et al., 2015). Thus, the PAL obstructed the transmission of light more significantly than MMT.

## Conclusions

The effects of 1D PAL nanorods and 2D MMT nanoplatelets on the mechanical, water resistance, and light transmission properties of the SA/PAL and SA/MMT nanocomposites were studied by a comparative study. It was revealed that PAL can enhance the mechanical and water resistance properties of SA/PAL nanocomposites better than MMT, because the 1D PAL has better dispersion than 2D MMT in the SA matrix at the same loading amounts. As a result of it, the TS of SA film increased sharply by 84.56% (from 13.67 to 25.23 MPa) after incorporation of 10 wt.% PAL, which is better than the TS of SA/MMT nanocomposite. In addition, the effect of PAL on the reduction of MU was more significant than that of MMT. Therefore, the 1D PAL nanorods are more suitable candidate of nanofillers to fabricate biopolymer-based nanocomposite films than MMT, and this study would lay a foundation to the design and preparation of new types of environment-friendly packing film materials.

## Author Contributions

DH and ZZ conceived and designed the experiments; ZM performed the experiments; DH, ZM, and ZZ analyzed the data; QQ contributed reagents, materials, and analysis tools; DH wrote the paper.

### Conflict of Interest Statement

The authors declare that the research was conducted in the absence of any commercial or financial relationships that could be construed as a potential conflict of interest.
